# Harnessing of inhomogeneously polarized Hermite–Gaussian vector beams to manage the 3D spin angular momentum density distribution

**DOI:** 10.1515/nanoph-2021-0418

**Published:** 2021-10-25

**Authors:** Svetlana N. Khonina, Aleksey P. Porfirev

**Affiliations:** Image Processing Systems Institute of RAS – Branch of the Federal Scientific Research Centre “Crystallography and Photonics” of Russian Academy of Sciences, Samara 443001, Russia; Samara National Research University, Samara 443086, Russia

**Keywords:** Hermite–Gaussian beams, polarization, spin angular momentum, Stokes vector, vector beams

## Abstract

We propose vector modes based on inhomogeneously polarized Hermite–Gaussian (HG) vector beams, providing complete structural conservation of the beams during propagation. Like uniformly polarized mode beams, these beams provide structural stability (or invariance) of both the intensity and the polarization state, in turn ensuring the stability of other field characteristics, including the angular momentum. We determine the conditions imposed on the HG mode composition in the transverse components of the electromagnetic field in order to control the three-dimensional characteristics of the field, such as intensity, polarization, and spin angular momentum (SAM). For the visual analysis of the polarization state of inhomogeneously polarized beams, we use the transverse distribution of the vector of three Stokes parameters. The correspondence of the third Stokes parameter to the distribution of the longitudinal component of the SAM is used for experimental measurements. The theoretical analysis is clearly illustrated by numerical simulations and confirmed by experimental results.

## 1 Introduction

The ability to control amplitude, phase, and polarization profiles of generated laser radiation allows one to shape light fields with the desired distributions of physical characteristics such as orbital and spin angular momentum (OAM and SAM) [1], [2], [3], [4]; real and imaginary parts of the complex Poynting vector density [5], [6], [7]; scattering, gradient, and other optical forces [8], [9], [10]; and many others [11], [12], [13], [14]. Controlling these characteristics is crucial for laser manipulation, laser material processing applications, and control of nanophotonics devices. Well-known examples of structured laser beams with controllable profiles include Laguerre–Gaussian (LG) and Hermite–Gaussian (HG) laser modes [15]. These laser modes represent mutually orthogonal modes with radial (LG modes) and rectangular (HG modes) symmetry. Interestingly, these two laser modes are related, and LG modes can be transformed into HG modes and vice versa with the help of astigmatic laser mode converters [16, 17]. LG modes are more widespread because higher-order LG modes have a nonzero total OAM that allows one to use them directly for the rotation of microparticles [18, 19] and indirectly for the spiral-shaped transfer of matter [20, 21]. The so-called astigmatic HG modes [22], as well as HG modes with embedded vortices [23], also have a nonzero OAM; however, they are not radially symmetric, which makes them difficult to use in several applications. The use of superpositions of LG or HG modes allows one to achieve an additional degree of freedom for controlling the profile of both the transverse and the longitudinal intensity and phase distributions [24], [25], [26]. This can lead, for example, to the generation of rotating light fields or light fields with periodically changing properties. In addition, the control of the polarization distribution of the superimposed modes makes it possible to generate cylindrical vector beams [27] and other types of inhomogeneously polarized vector beams [28]. The nonuniform polarization distribution of such light fields allows one to use them for the realization of pulling/pushing laser beams [29], polarization division multiplexing for optical communication [30], and the formation of laser-induced periodic surface structures with nonconventional morphologies [31].

Recently, much attention has been paid to the formation of light fields with the desired longitudinal and transverse SAM. Such light fields find applications in the field of laser manipulation for the realization of “photonic wheels” [32, 33], polarization knots [34, 35], and polarization ribbons [36, 37]. The realization of complex light patterns in the transverse polarization plane allows one to generate complex optical longitudinal polarization structures including linked and knotted longitudinal vortex lines [38], [39], [40] that are interesting from the point of view of nonlinear optics and Bose–Einstein condensates. Also, except for the three-dimensional (3D) distribution of light field characteristics in space (which is considered in this work), there is a possibility of local 3D directional control of the field, in particular, the polarization state or SAM can be modulated at local points of space in different directions [41], [42], [43], [44], [45] and may be exploited to observe unusual properties of light such as the Belinfante’s spin momentum which was previously considered just theoretically.

It is well known that in the case of linearly polarized radiation, the total SAM and the SAM density are zero at each point. For left or right circularly polarized radiation, each of its photons carries a SAM defined as ±*ħ* per photon (where *ħ* is the reduced Planck constant) and a SAM density that is proportional to the intensity. Elliptical polarization can be written as a superposition of left and right circular polarizations, and then the SAM depends on the relative magnitudes of the combination of the two circular polarization states. However, in the case of nonuniformly polarized radiation, the situation is completely different, with a zero total SAM; however, the local distribution of the SAM density is not uniform. For example, recently, the new class of nonuniform beams, so-called vector Lissajous beams (VLBs) [28], was introduced, for which the possibility of controlling the transverse SAM density was demonstrated. VLBs were formed using an interferometric approach for the summation of two orthogonally polarized laser modes.

Here, we present a similar approach for the generation of inhomogeneously polarized HG vector beams, the transverse components of which are different HG modes or superpositions of HG modes. When the HG modes indices are selected in accordance with certain conditions, it is possible to form inhomogeneously polarized vector modes that preserve not only the transverse structure (intensity distribution up to scale, as with scalar laser modes) but also the polarization state when the vector beams propagate in free space. In a recent paper [46], a similar approach to the formation of vector beams was considered for various Gaussian beams and their propagation properties were analyzed based on the Gouy phase. However, the possibility of managing 3D SAM density distribution was not considered.

The ability to form inhomogeneously polarized vector modes expands the capabilities of laser beams in various applications, such as laser structuring and trapping and manipulating microparticles. Since the properties of the generated vector laser beams are defined by the superpositions of HG modes, we can manage the SAM density distribution in 3D space as the beams are propagating. We believe that this possibility can be used not only in optical manipulation applications but also in the field of spintronics for the creation and mapping of a spin current in materials [47]. Effective generation of spin currents is crucial in spintronics applications [48, 49], and photoexcitation using laser radiation with SAM is one of the methods for this [50].

In this study, we are interested in the structure of the transverse distribution of various field characteristics and its change during beam propagation in free space, therefore, significant attention is paid to an understandable and convenient visualization of the considered characteristics. We used the transverse distribution of the vector of three Stokes parameters for the visual analysis of the polarization state of inhomogeneously polarized beams similar to the visualization of field intensity with three components. The correspondence of the third Stokes parameter to the distribution of the longitudinal component of the SAM is used for experimental measurements. The theoretical analysis is clearly illustrated by numerical simulations and confirmed by experimental results.

## 2 Theoretical analysis

### 2.1 Conditions for the generation of inhomogeneously polarized HG vector modes

The propagation of vector beams in free space can be described using the Rayleigh–Sommerfeld integral [51], [52], [53]:

(1) $$E\left(u,v,z\right)=\left(\begin{array}{l} E_{x}\left(u,v,z\right)\\ E_{y}\left(u,v,z\right)\\ E_{z}\left(u,v,z\right) \end{array}\right)=\frac{1}{2\pi }\iint \left(\begin{array}{l} zE_{0x}\left(x,y\right)\\ zE_{0y}\left(x,y\right)\\ -\left[E_{0x}\left(x,y\right)\left(u-x\right)+E_{0y}\left(x,y\right)\left(v-y\right)\right] \end{array}\right)\frac{{\text{e}}^{ik\ell }}{\ell^{2}}\left(ik-\frac{1}{\ell }\right)\text{ d}x\text{d}y\text{,}$$

where *E*_0*x*_(*x*, *y*) and *E*_0*y*_(*x*, *y*) are the complex amplitudes of the *x*- and *y*-components of the input electric field, $\ell ={\left[{\left(u-x\right)}^{2}+{\left(v-y\right)}^{2}+z^{2}\right]}^{1/2}$ , k=2π/λ is the wavenumber, and *λ* is the wavelength.

In the paraxial case, $\ell \approx z+\left[{\left(u-x\right)}^{2}+{\left(v-y\right)}^{2}\right]/2z$ , and Eq. (1) is substantially simplified. In addition, since the optical axis has been chosen to be *z*, and the initial field only has *x* and *y* components, the contribution of the longitudinal component is small; therefore, it makes sense to consider only the transverse components of the field. Then Eq. (1) takes the following form (which is the Fresnel diffraction integral):

(2) $$E_{⊥}\left(u,v,z\right)=\left(\begin{array}{l} E_{x}\left(u,v,z\right)\\ E_{y}\left(u,v,z\right) \end{array}\right)=\frac{ik}{2\pi z}\exp\left(ikz\right)\iint \left(\begin{array}{l} E_{0x}\left(x,y\right)\\ E_{0y}\left(x,y\right) \end{array}\right)\exp\left[ik\frac{{\left(u-x\right)}^{2}+{\left(v-y\right)}^{2}}{2z}\right]\text{ d}x\text{d}y\text{.}$$

Let us consider the situation in which the transverse electric components $E_{0x}\left(x,y\right)\text{, }E_{0y}\left(x,y\right)$ of the input field consist of HG modes [15, 54]:

(3) $$\Psi_{nm}\left(x,y\right)=\exp\left(-\frac{x^{2}+y^{2}}{2\sigma^{2}}\right)H_{n}\left(\frac{x}{\sigma }\right)H_{m}\left(\frac{y}{\sigma }\right)\text{,}$$

where *σ* is a Gaussian parameter and *H*_*n*_(*x*) is an *n*-order Hermite polynomial. For *n* = *m* = 0, the beam in Eq. (3) corresponds to the fundamental Gaussian beam, so the width of the beam is equal to $w=\sqrt{2}\sigma \text{.}$

It is known [54, 55] that HG modes that propagate in free space retain their structure but change their scale, acquiring a phase shift in accordance with the mode indices:

(4) $$\begin{array}{l} \Psi_{nm}\left(u,v,z\right)=\frac{\sigma_{0}}{\sigma \left(z\right)}\exp\left[i\left(n+m+1\right)\;\eta \left(z\right)\right]\times \\ \times \exp\left[\frac{-i\pi \left(u^{2}+v^{2}\right)}{\lambda R\left(z\right)}\right]\exp\left[-\frac{u^{2}+v^{2}}{2\sigma^{2}\left(z\right)}\right]H_{n}\left(\frac{u}{\sigma \left(z\right)}\right)H_{m}\left(\frac{v}{\sigma \left(z\right)}\right)\text{,} \end{array}$$

where $\eta \left(z\right)=\text{arctg }\left(z/z_{0}\right)$ , $R\left(z\right)=z\left(1+z_{0}^{2}/z^{2}\right)$ , $\sigma \left(z\right)=\sigma_{0}{\left(1+z^{2}/z_{0}^{2}\right)}^{1/2}$ , and $z_{0}=2\pi \sigma_{0}^{2}/\lambda$ is the Rayleigh distance.

Taking into account the structural invariance of the HG modes during propagation, we can write Eq. (4) in the following form:

(5) $$\Psi_{nm}\left(x,y,z\right)=\exp\left[i\left(n+m+1\right)\eta \left(z\right)\right]\Phi_{nm}\left(x,y;z\right)=\gamma_{nm}\left(z\right)\Phi_{nm}\left(x,y;z\right)\text{,}$$

where $\Phi_{nm}\left(x,y;z\right)$ are functions that contain common components for all modes, as well as components that do not affect the change in the state of polarization, and $\gamma_{nm}\left(z\right)$ are eigenvalues:

(6) $$\gamma_{nm}\left(z\right)=\exp\left[i\left(n+m+1\right)\eta \left(z\right)\right]\text{,}$$

which determine the phase shift for each individual HG mode depending on the indices (*n*, *m*) and the propagated distance *z*.

Then the vector field defined by Eq. (3) can be written as follows:

(7) $$E_{⊥}\left(u,v,z\right)=\left(\begin{array}{l} \Psi_{nm}\left(u,v,z\right)\\ \Psi_{pq}\left(u,v,z\right) \end{array}\right)=\left(\begin{array}{l} \gamma_{nm}\left(z\right)\Phi_{nm}\left(u,v;z\right)\\ \gamma_{pq}\left(z\right)\Phi_{pq}\left(u,v;z\right) \end{array}\right)\text{.}$$

Since the eigenvalues $\gamma_{nm}\left(z\right)$ express only the phase shift, the total field intensity defined by Eq. (7) can be described by the following expression:

(8) $${\left|E_{⊥}\left(u,v,z\right)\right|}^{2}={\left|\gamma_{nm}\left(z\right)\right|}^{2}{\left|\Phi_{nm}\left(u,v;z\right)\right|}^{2}+{\left|\gamma_{pq}\left(z\right)\right|}^{2}{\left|\Phi_{pq}\left(u,v;z\right)\right|}^{2}={\left|\Phi_{nm}\left(u,v;z\right)\right|}^{2}+{\left|\Phi_{pq}\left(u,v;z\right)\right|}^{2}\text{.}$$

From Eq. (8) it follows that the beam intensity does not depend on the polarization state and, taking into account the properties of the HG modes, will be structurally unchanged up to scale. However, the polarization state of such a beam will change depending on the propagation distance *z* and the HG mode indices:

(9) $$\left(\begin{array}{l} c_{x}\left(z\right)\\ c_{y}\left(z\right) \end{array}\right)=\gamma_{nm}\left(z\right)\left(\begin{array}{l} 1\\ \frac{\gamma_{pq}\left(z\right)}{\gamma_{nm}\left(z\right)} \end{array}\right)=\gamma_{nm}\left(z\right)\left(\begin{array}{l} 1\\ \exp\left\{i\left[\left(n+m\right)-\left(p+q\right)\right]\eta \left(z\right)\right\} \end{array}\right)\text{.}$$

As follows from Eq. (9), for vector beams, the sum of their indices is the same:

(10) (n+m)=(p+q),

so the polarization state will not change. That is, such a field can be called an inhomogeneously polarized vector mode. In other cases, the state of polarization will change in a certain way.

Obviously, by maintaining the condition defined by Eq. (10) for several (more than two) modes, more complex inhomogeneously polarized vector modes can be formed:

(11) $$E_{⊥}\left(u,v,z\right)=\left(\begin{array}{l} \sum_{n,m\in \Omega }b_{nm}\Psi_{nm}\left(u,v,z\right)\\ \sum_{p,q\in \Omega }b_{pq}\Psi_{pq}\left(u,v,z\right) \end{array}\right)\text{,}$$

where $b_{nm}$ are arbitrary complex coefficients and Ω is the set of indices satisfying the condition of equality of their sum:

(12) Ω:n+m=const.

Note that a similar approach was considered in Ref. [42] for uniformly polarized multimode HG beams. In particular, for a beam consisting of two HG modes, the intensity has the following form:

(13) $${\left|E_{⊥}\left(u,v,z\right)\right|}^{2}={\left|\Phi_{nm}\left(x,y;z\right)\right|}^{2}+{\left|\Phi_{pq}\left(x,y;z\right)\right|}^{2}+2\text{Re}\left[\Phi_{nm}\left(x,y;z\right)\Phi_{pq}^{*}\left(x,y;z\right)\right]\cos\left\{\left[\left(n+m\right)-\left(p+q\right)\right]\eta \left(z\right)\right\}\text{.}$$

Obviously, the beam will be structurally invariant under the condition defined by Eq. (10). Otherwise, the intensity will periodically change in accordance with the traveled distance *z* and the ratio of the mode indices.

In Ref. [56], the condition of periodic self-reproduction of the intensity of a multimode HG beam at various distances from the optical element was also obtained. In particular, the intensity distribution formed at a distance *z*_*s*_ will be repeated at the following distance:

(14) $$z_{l}=\frac{z_{s}+z_{0}\text{ tan}\left(2\pi l/d\right)}{1-z_{s}\text{ tan}\left(2\pi l/d\right)/z_{0}}\text{,}$$

where d=(n+m)−(p+q) and *z*_0_ is the Rayleigh distance.

For the input field (*z*_*s*_ = 0), Eq. (14) is greatly simplified:

(15) $$z_{l}=z_{0}\text{ tan}\left(2\pi l/d\right)\text{.}$$

The index *l* in Eqs. (14) and (15) means that there can be several distances of periodic repetition. In principle, the number of repetitions increases with the growth in the difference *d* between the indices. If *d* = 0, then the beam will be structurally stable (invariant) during propagation.

### 2.2 Characteristics of HG vector beams

The SAM density distribution is calculated by the following formula [57]:

(16) $$s∼\text{Im}\left(\epsilon E^{*}\times E+\mu H^{*}\times H\right)\text{.}$$

Often only the electrical part of the SAM is considered:

(17) $$s_{E}∼\text{Im}\left(E^{*}\times E\right)=\text{Im}\left(\begin{array}{l} E_{y}^{*}E_{z}-E_{z}^{*}E_{y}\\ E_{z}^{*}E_{x}-E_{x}^{*}E_{z}\\ E_{x}^{*}E_{y}-E_{y}^{*}E_{x} \end{array}\right)\text{.}$$

In the paraxial case, the longitudinal component of the SAM dominates the transverse components. Let us consider this in more detail:

(18) $$s_{E,z}≃\text{Im}\left[E_{x}^{*}E_{y}-E_{y}^{*}E_{x}\right]\text{.}$$

For uniformly polarized beams, Eq. (18) is completely determined by the polarization state, i.e. by components of the polarization vector ${\left(c_{x},c_{y}\right)}^{T}$ :

(19) $$s_{E,z}≃\text{Im}\left[{\left|E_{⊥}\right|}^{2}\left(c_{x}^{*}c_{y}-c_{y}^{*}c_{x}\right)\right]\text{,}$$

where ${\left|E_{⊥}\right|}^{2}$ is the field intensity in the considered transverse plane.

Obviously, for a linearly polarized field (with real components of the polarization vector), $s_{E,z}^{\text{lin}}=0$ , and for a field with a circular “±”-polarization $c_{y}=\pm ic_{x}$ (*c*_*x*_ is real), Eq. (19) is reduced to the following:

(20) $$s_{E,z}^{\text{cir}\pm }≃\text{Im}\left[{\left|E_{⊥}\right|}^{2}\left(c_{x}\left(\pm ic_{x}\right)-\left(\mp ic_{x}\right)c_{x}\right)\right]=\text{Im}\left[\pm 2ic_{x}^{2}{\left|E_{⊥}\right|}^{2}\right]=\pm 2c_{x}^{2}{\left|E_{⊥}\right|}^{2}\text{.}$$

Thus, for a circularly polarized field, the SAM density of the longitudinal component is proportional to the field intensity. If the field intensity retains its structure during propagation, then the SAM density distribution will also remain the same. This property may be useful in various applications, such as laser structuring and trapping and manipulating microparticles.

For nonuniformly polarized beams, the characteristics of a field defined by Eq. (11) are completely determined by the HG mode composition in the transverse components of the field. For the considered HG vector beams defined by Eq. (11), the general expression of the SAM density defined by Eq. (18) can be written explicitly:

(21) $$s_{E,z}≃\text{Im}\left[E_{x}^{*}E_{y}-E_{y}^{*}E_{x}\right]=2\text{Im}\left[E_{x}^{*}E_{y}\right]=2\text{Im}\left[{\left(\sum_{n,m\in \Omega }b_{nm}\Psi_{nm}\right)}^{*}\left(\sum_{p,q\in \Omega }b_{pq}\Psi_{pq}\right)\right]\text{.}$$

Taking into account that the HG modes defined by Eq. (4) are orthogonal, the total SAM for nonuniformly polarized HG beams defined by Eq. (11) will be equal to zero. However, nonzero SAM density values can be used to control locally trapped particles [58], [59], [60].

By imposing conditions on the mode indices in the superposition, it is possible to control the 3D characteristics of the field. In particular, it is possible to change the intensity or polarization state of the beam during propagation (for example, periodic or stable). This, in turn, can change or stabilize other characteristics of the field, including the SAM. In this work, by the term “stability” we mean the preservation of the beam structure (intensity, polarization state, or SAM distribution) up to scale during propagation in free space.

For nonuniformly polarized beams, the distribution of the polarization state is a very important characteristic. However, for complex polarized beams, the state of polarization can change noticeably even at neighboring points, so it is difficult to use classical visualization methods in the form of arrows. Therefore, in this work, for clear visualization of the polarization distribution, we also use the distributions of the Stokes parameters [61, 62]:

(22) $$\begin{array}{l} S_{0}\left(x,y\right)={\left|E_{x}\left(x,y\right)\right|}^{2}+{\left|E_{y}\left(x,y\right)\right|}^{2}=I_{0{}^{\circ}}+I_{90{}^{\circ}},\\ S_{1}\left(x,y\right)={\left|E_{x}\left(x,y\right)\right|}^{2}-{\left|E_{y}\left(x,y\right)\right|}^{2}=I_{0{}^{\circ}}-I_{90{}^{\circ}},\\ S_{2}\left(x,y\right)=2\text{Re}\left[E_{x}^{*}\left(x,y\right)E_{y}\left(x,y\right)\right]=2\left|E_{x}\left(x,y\right)\right|\left|E_{y}\left(x,y\right)\right|\cos\left[\delta \left(x,y\right)\right]=I_{45{}^{\circ}}-I_{135{}^{\circ}},\\ S_{3}\left(x,y\right)=2\text{Im}\left[E_{x}^{*}\left(x,y\right)E_{y}\left(x,y\right)\right]=2\left|E_{x}\left(x,y\right)\right|\left|E_{y}\left(x,y\right)\right|\sin\left[\delta \left(x,y\right)\right]=I_{R\left(-\right)}-I_{L\left(+\right)}, \end{array}$$

where δ(x,y) is the phase difference between the *x*- and *y*-components of the vector field.

For the paraxial case, $S_{0}\left(x,y\right)$ corresponds to ${\left|E_{⊥}\right|}^{2}$ , and the polarization characteristics of the field reflect the rest of the Stokes parameters. For uniformly polarized beams, the Stokes parameters are completely determined by the initial state of polarization, i.e. the components of the polarization vector ${\left(c_{x},c_{y}\right)}^{T}\text{.}$ In this case, instead of Eq. (22), we can write

(23) $$\begin{array}{l} S_{0}\left(x,y\right)={\left|E_{⊥}\right|}^{2}\left({\left|c_{x}\right|}^{2}+{\left|c_{y}\right|}^{2}\right)={\left|E_{⊥}\right|}^{2},\\ S_{1}\left(x,y\right)={\left|E_{⊥}\right|}^{2}\left({\left|c_{x}\right|}^{2}-{\left|c_{y}\right|}^{2}\right),\\ S_{2}\left(x,y\right)=2{\left|E_{⊥}\right|}^{2}\left|c_{x}\right|\left|c_{y}\right|\cos\left[\delta \left(x,y\right)\right],\\ S_{3}\left(x,y\right)=2{\left|E_{⊥}\right|}^{2}\left|c_{x}\right|\left|c_{y}\right|\;\sin\left[\delta \left(x,y\right)\right]\text{.} \end{array}$$

As can be seen from Eq. (23), for uniformly polarized beams, all Stokes parameters are proportional to the total field intensity, and, depending on the polarization vector ${\left(c_{x},c_{y}\right)}^{T}$ , some of the parameters are completely zero. For example, for horizontally polarized (*c*_*y*_ = 0) or vertically polarized (*c*_*x*_ = 0) fields, $S_{2}\left(x,y\right)=S_{3}\left(x,y\right)=0$ ; for diagonally polarized ( $\left|c_{y}\right|=\left|c_{x}\right|$ , δ(x,y)=±π ) fields, $S_{1}\left(x,y\right)=S_{3}\left(x,y\right)=0$ ; and for circularly polarized ( $\left|c_{y}\right|=\left|c_{x}\right|$ , δ(x,y)=±π/2 ) fields, $S_{1}\left(x,y\right)=S_{2}\left(x,y\right)=0\text{.}$

Thus, for uniformly polarized fields, the three Stokes parameters normalized to the intensity are conveniently associated with the coordinates of various points on the Poincare sphere [63, 64].

However, for nonuniformly polarized beams, the distribution of the Stokes parameters will be much more complicated, since at different points of the field there will be different states of polarization.

Some studies have considered various generalizations of the Poincare sphere [65], [66], [67], [68] and use advanced visualization techniques for vector beam states [69], [70], [71]; however, for complex polarization distributions, it is more convenient to use a different visualization method, in the form of color halftones for the Stokes vector:

(24) $$St\left(u,v,z\right)=\left(\begin{array}{l} S_{1}\left(u,v,z\right)\\ S_{2}\left(u,v,z\right)\\ S_{3}\left(u,v,z\right) \end{array}\right)\text{.}$$

This representation is also convenient for visualizing polarization singularities [72, 73].

Note that there is a complete correspondence between the longitudinal component of the SAM density and the distribution of the third Stokes parameter [74, 75]:

(25) $$s_{E,z}≃\text{Im}\left[E_{x}^{*}E_{y}-E_{y}^{*}E_{x}\right]=2\text{Im}\left[E_{x}^{*}E_{y}\right]≃S_{3}\text{.}$$

This fact is important for experimental measurements.

Various examples of HG vector modes are discussed in the following sections.

Note that, as a rule, the normalized Stokes parameters are considered. However, in this work, we are interested in the relationship between the third Stokes parameter and the SAM density expressed by Eq. (25), as well as the dynamics of this characteristic change during field propagation in free space. Therefore, further in this work, we use the normalization (see digits on the scale-bars) to the global maximum value of the considered characteristic in the considered 3D region.

## 3 Simulation results

The following parameter values were used in the simulation: the size of the input field is 3 mm × 3 mm, the wavelength of the illuminating beam is *λ* = 533 nm, and $\sigma_{0}=0.5\text{ mm.}$ For the considered parameter values, the Rayleigh distance is $z_{0}=2\pi \sigma_{0}^{2}/\lambda \approx 3000\text{ mm.}$

To show the transverse intensity in colors we use the ratio ${\left|E_{⊥}\right|}^{2}={\left|E_{x}\right|}^{2}+{\left|E_{y}\right|}^{2}$ : red color for ${\left|E_{x}\right|}^{2}$ and green color for ${\left|E_{y}\right|}^{2}$ (for the uniform polarization at ${\left|E_{x}\right|}^{2}$ = ${\left|E_{y}\right|}^{2}$ a yellow color is obtained). The structure of the SAM density, $s_{E,z}\left(u,v,z\right)$ , calculated using Eq. (21) is shown by blue color for positive values of $s_{E,z}\left(u,v,z\right)$ and pink color for negative values. We also show polarization state by the amplitude distribution of the Stokes vector $\left|St\right|=\left|S_{1}\right|+\left|S_{2}\right|+\left|S_{3}\right|$ (by analogy with visualizing the total field intensity as the sum of the intensities of the individual components ${\left|E\right|}^{2}={\left|E_{x}\right|}^{2}+{\left|E_{y}\right|}^{2}+{\left|E_{z}\right|}^{2}$ ) in colors: turquoise color for $\left|S_{1}\right|$ , crimson color for $\left|S_{2}\right|$ , and yellow color for $\left|S_{3}\right|\text{.}$

The digits on the scale-bars for $s_{E,z}\left(u,v,z\right)$ are given in accordance with the normalization to the global maximum value in the considered 3D region: u,v∈[−2 mm,2 mm] , z∈[0,3000 mm].

### 3.1 Periodic HG vector beams

Let us first consider the propagation of uniformly polarized (with “+”-circular polarization) two-mode HG beams with indices (*n*, *m*) and (*p*, *q*). Such a beam has the following distribution in the input plane:

(26) $$E_{0}\left(x,y\right)=\frac{1}{\sqrt{2}}\left(\Psi_{\left(n,m\right)}\left(x,y\right)+\Psi_{\left(p,q\right)}\left(x,y\right)\right)\left(e_{x}+ie_{y}\right)\text{.}$$

**Figure 1:**
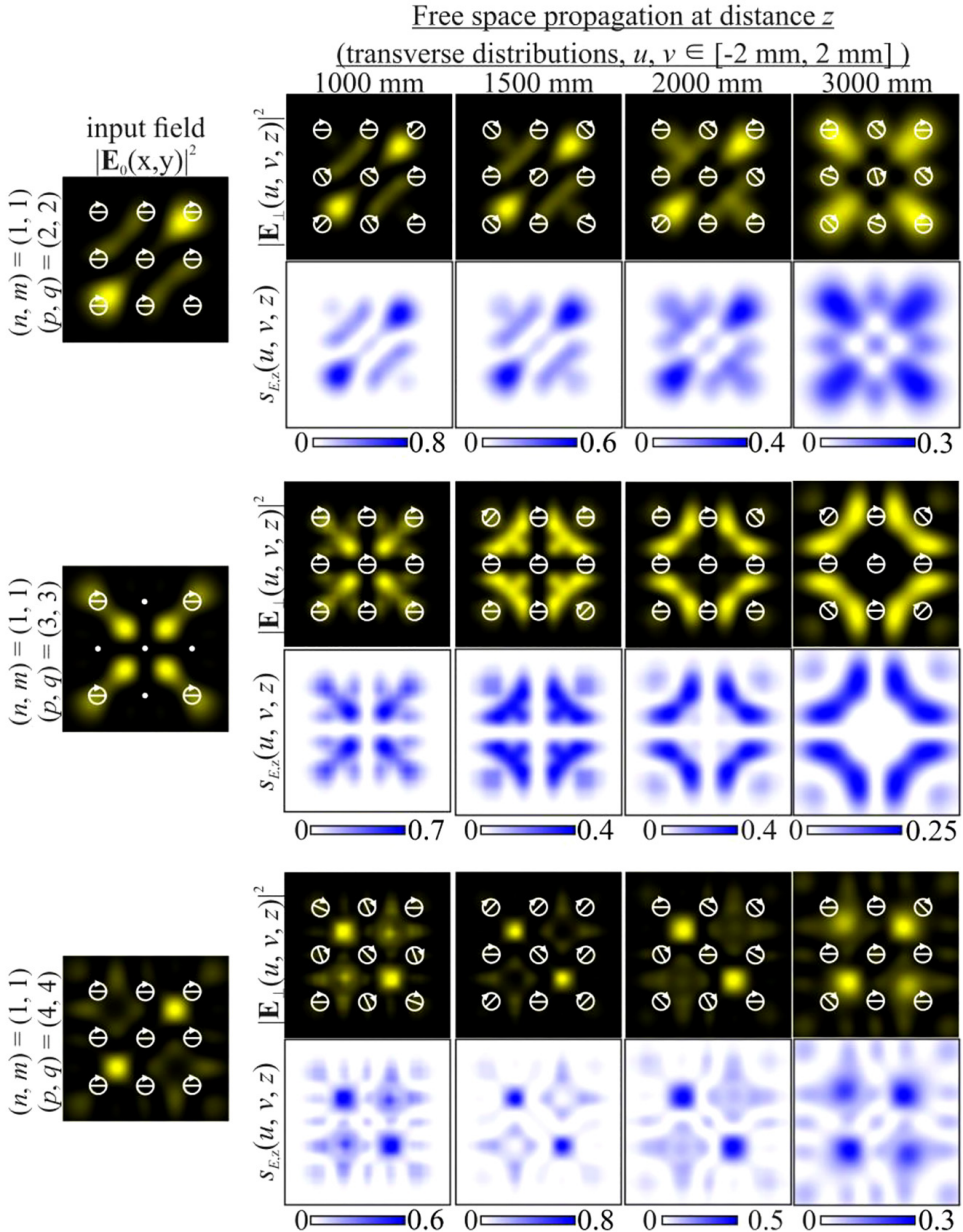
Propagation of uniformly polarized (“+”-circular polarization) two-mode HG beams defined by Eq. (26) with indices (*n* + *m*) ≠ (*p* + *q*).

Figure 1 shows the simulation results for the beam described by Eq. (26) with indices (n+m)≠(p+q). In this case, the field intensity will not be conserved during propagation [56], although the polarization state will remain unchanged. If the difference between the indices is small – for example, for (*n*, *m*) = (1, 1) and (*p*, *q*) = (2, 2), we get *d* = 2 (first line of Figure 1), or for (*n*, *m*) = (1, 1) and (*p*, *q*) = (3, 3), we get *d* = 4 (second row of Figure 1) – then using Eq. (15) we calculate that $z_{1}=\infty$ ; i.e. even the input field distribution (*z*_*s*_ = 0) will never be repeated. If we increase the difference between the indices – for example, for (*n*, *m*) = (1, 1) and (*p*, *q*) = (4, 4), *d* = 6 – then the input field distribution will be repeated with a scale at $z_{1}\approx 4000\text{ mm}$ (third line of Figure 1).

As can be seen from the results shown in Figure 1, the distribution of the longitudinal component of the SAM, $s_{E,z}\left(u,v,z\right)$ , is the same as the distribution of the field intensity at different distances and changes accordingly. This is in complete agreement with the theoretical analysis performed in the previous section. Obviously, it is possible to form various field structures and SAM distributions. Note that while the value of the total (integral) SAM is conserved, the SAM density distribution can change significantly, including the local maximum values (the digits on the scale-bars are normalized to the global maximum value in the considered 3D region).

If an inhomogeneously polarized beam is formed in accordance with Eq. (7), then the input field will take the following form:

(27) $$E_{0}\left(x,y\right)=\frac{1}{\sqrt{2}}\left(\begin{array}{l} \Psi_{\left(n,m\right)}\left(x,y\right)\\ a\Psi_{\left(p,q\right)}\left(x,y\right) \end{array}\right)=\frac{1}{\sqrt{2}}\left(\Psi_{\left(n,m\right)}\left(x,y\right)e_{x}+a\Psi_{\left(p,q\right)}\left(x,y\right)e_{y}\right)\text{,}$$

where *a* is a complex constant that determines the initial phase shift between the transverse field components. Since HG modes are real functions, the constant *a* defines the initial polarization state.

**Figure 2:**
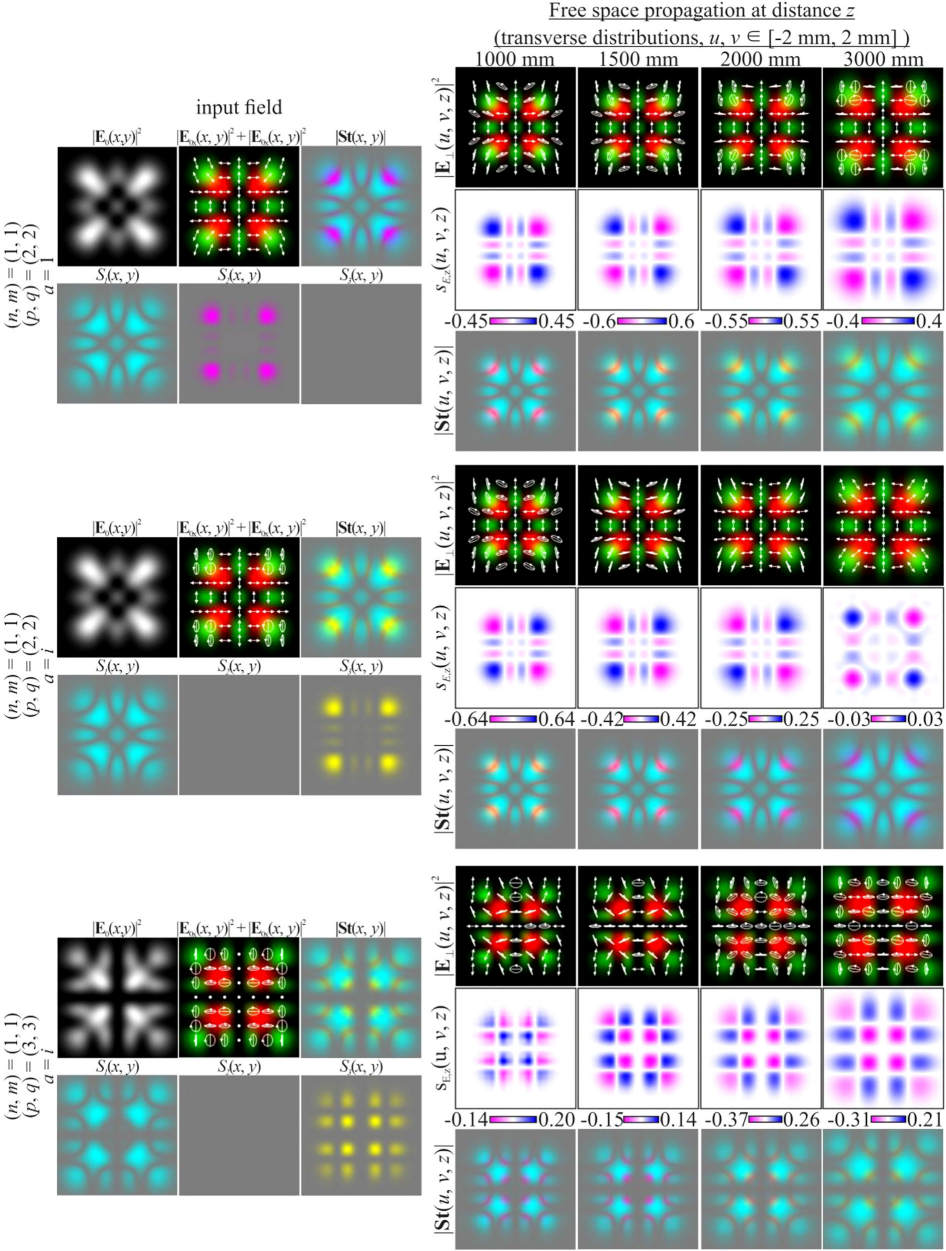
Propagation of inhomogeneously polarized beams defined by Eq. (27) with indices (*n* + *m*) ≠ (*p* + *q*) at different initial phase shifts *a* between the transverse components of the field.

In this case, the intensity of the beam during propagation retains the initial picture up to scale, but the state of the initial polarization changes (white arrows in Figure 2). Although the structure of the SAM density is preserved in this case, its magnitude changes significantly, since there is a local change in the polarization state (for example, from linear to elliptical or vice versa).

The change in polarization for an inhomogeneously polarized beam can be seen more clearly from the distribution of the Stokes vector defined by Eq. (24). Figure 2 shows the amplitude distributions of the Stokes vector $\left|St\right|=\left|S_{1}\right|+\left|S_{2}\right|+\left|S_{3}\right|$ . Note that zero lines of $\left|S_{1}\right|$ are clearly visible, which correspond to regions with diagonal linear polarization, as well as regions with circular polarization (one of the types of polarization singularity [73, 74]). As seen from Figure 2, the distribution of the SAM density and *S*_3_ are identical in structure (we show $\left|S_{3}\right|$ in this section). When the initial phase shift between the transverse components of the field is equal to zero (*a* = 1), $S_{3}\left(x,y\right)=0$ , and when *a* = *i* (the initial phase shift is equal to *π*/4), $S_{2}\left(x,y\right)=0\text{.}$

### 3.2 Stable HG vector beams (vector modes)

To generate vector modes, i.e. beams that preserve both the intensity pattern and the state of polarization during propagation, it is necessary to fill the transverse components of the vector field by HG modes with indices satisfying the condition defined by Eq. (10).

**Figure 3:**
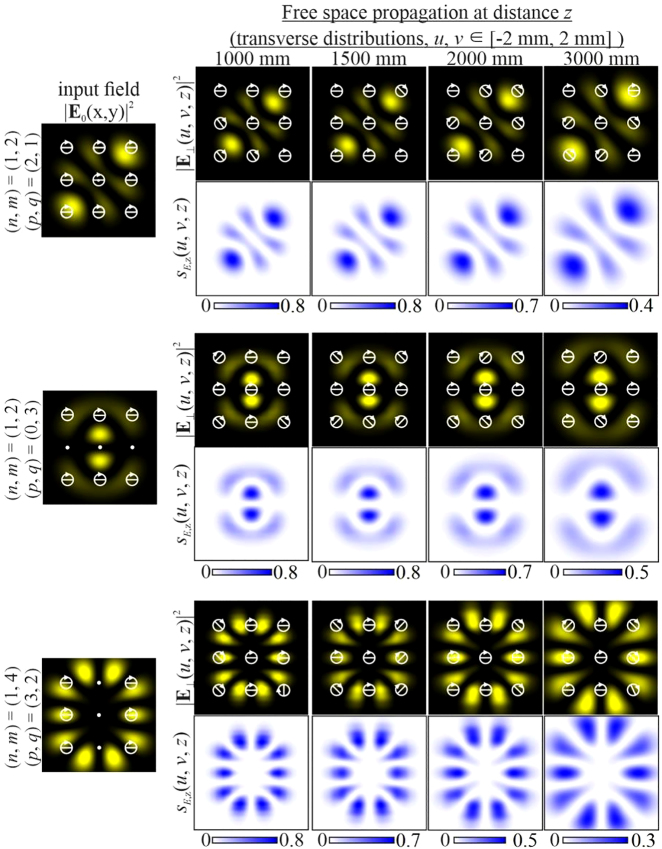
Propagation of uniformly polarized (“+”-circular polarization) two-mode HG beams defined by Eq. (26) with indices (*n* + *m*) = (*p* + *q*) (uniformly polarized vector modes).

Let us first consider uniformly polarized two-mode HG beams, the input field of which is described by Eq. (26) with indices (n+m)=(p+q). Figure 3 shows various examples of such beams. As can be seen, the fields retain not only the initial state of polarization, which is expected for paraxial propagation but also the intensity pattern, which changes only in scale, as with individual HG modes.

Since the SAM density distribution for circularly polarized beams is determined by the field intensity in the transverse plane, in this case, the structural stability or invariance (up to scale) of the SAM density of various configurations is ensured. The decrease in absolute values is associated with a scale broadening of the structure (the increase in area is compensated by a decrease in absolute values to maintain a constant value of the total SAM).

**Figure 4:**
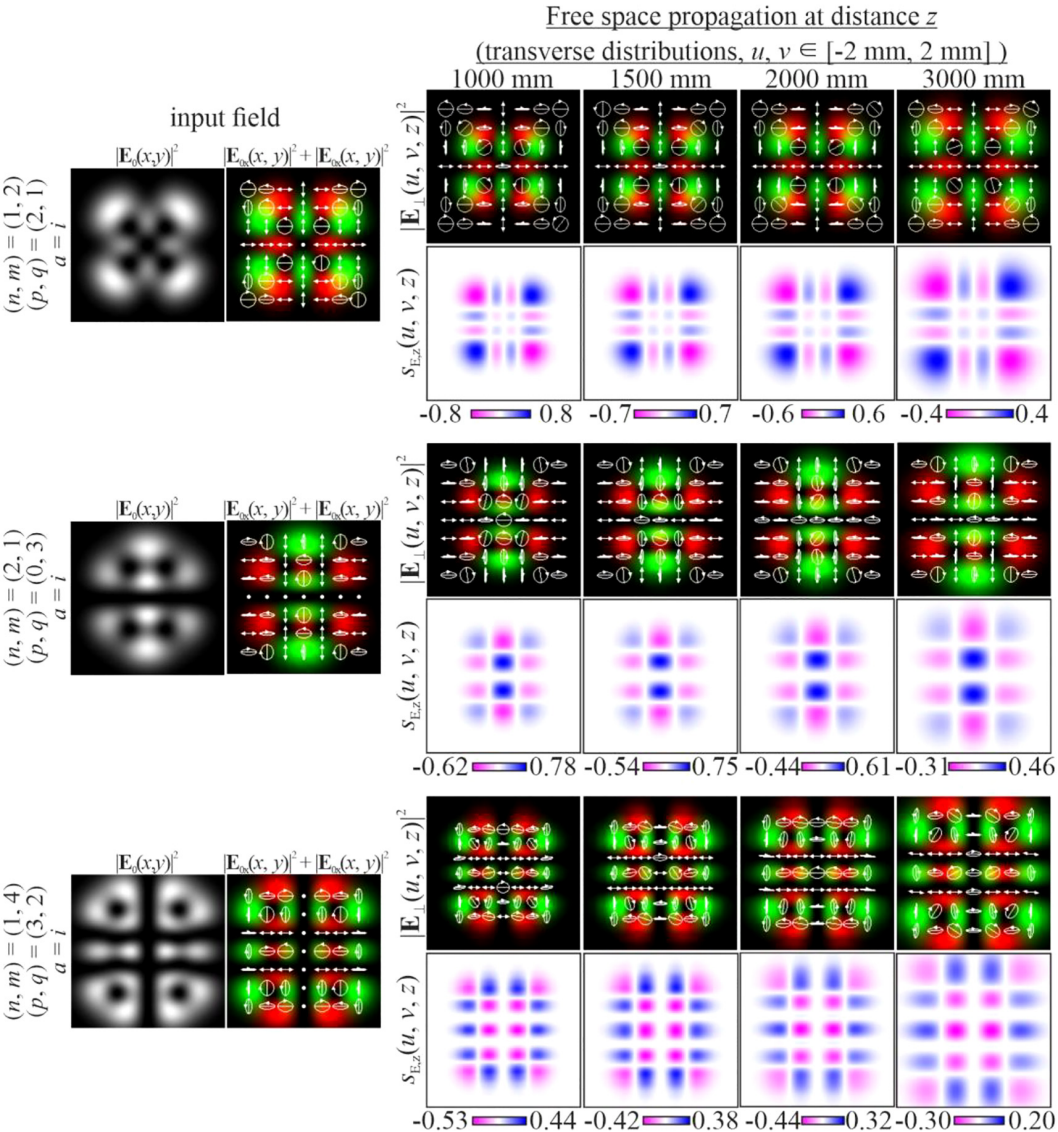
Propagation of inhomogeneously polarized beams defined by Eq. (27) with indices (*n* + *m*) = (*p* + *q*) (nonuniformly polarized vector modes) at the initial phase shift *a* = *i*.

**Figure 5:**
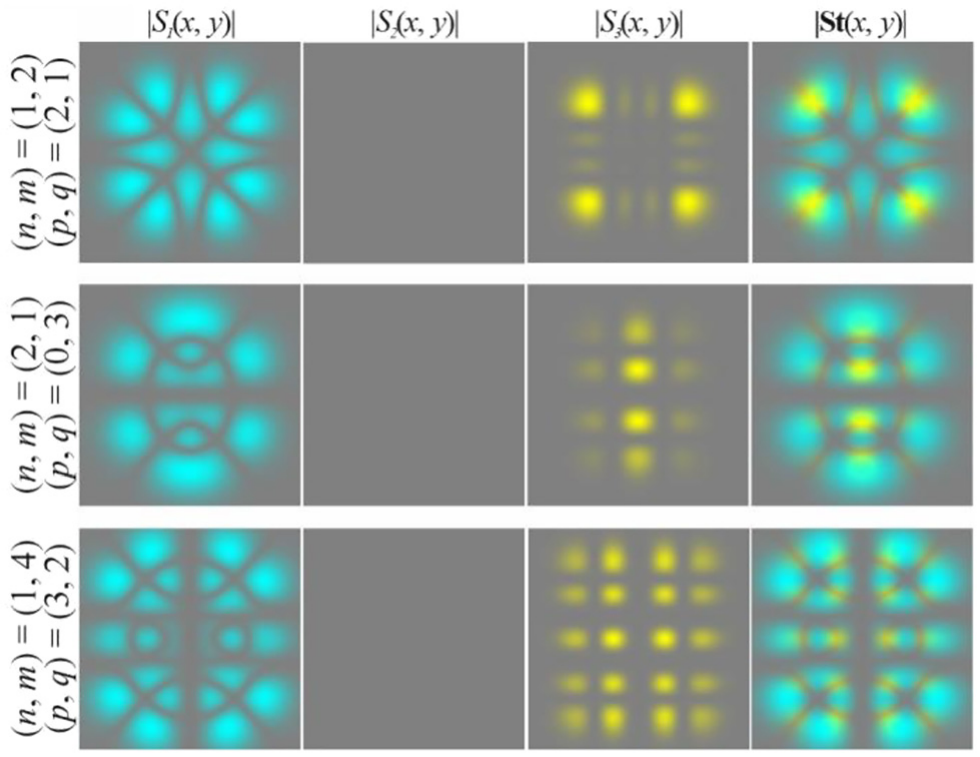
Distribution of the Stokes vector defined by Eq. (24) for inhomogeneously polarized beams defined by Eq. (27) presented in Figure 4.

Next, we consider inhomogeneously polarized HG beams defined by Eq. (27) with indices (n+m)=(p+q). In particular, Figure 4 shows the simulation results for different pairs of indices (*n*, *m*) and (*p*, *q*) satisfying the condition defined by Eq. (10). As can be seen, the fields preserve not only the intensity pattern up to scale but also the initial state of polarization (the absolute values of the SAM density change because of scale broadening). Note that in this case, both the intensity distribution and the polarization state differ significantly from those of the field defined by Eq. (26) (compare the first lines of Figures 3 and 4). Figure 5 shows the corresponding distributions of the amplitudes of the Stokes vector components defined by Eq. (24) in the input plane (during propagation, these distributions are preserved up to scale). $S_{2}\left(x,y\right)=0$ because the initial phase shift *a* between the transverse components of the field is equal to *i*.

**Figure 6:**
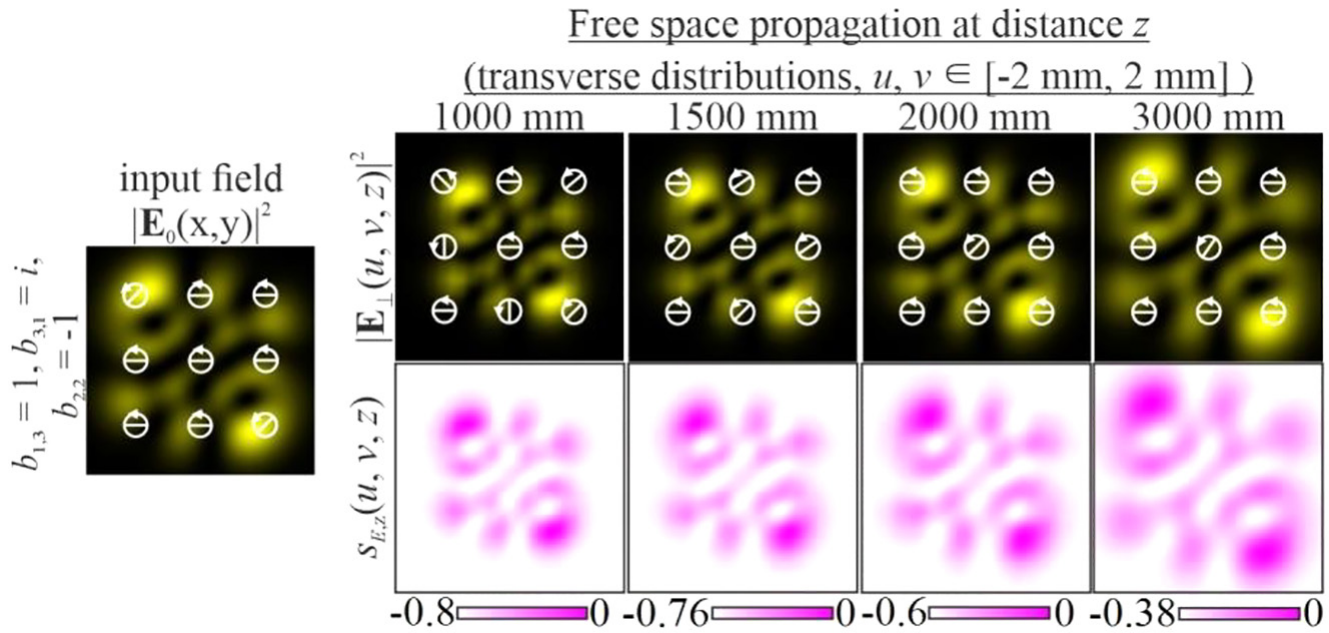
Propagation of a circularly polarized multimode HG beam defined by Eq. (11) with indices satisfying the condition defined by Eq. (12) (a uniformly polarized vector mode).

**Figure 7:**
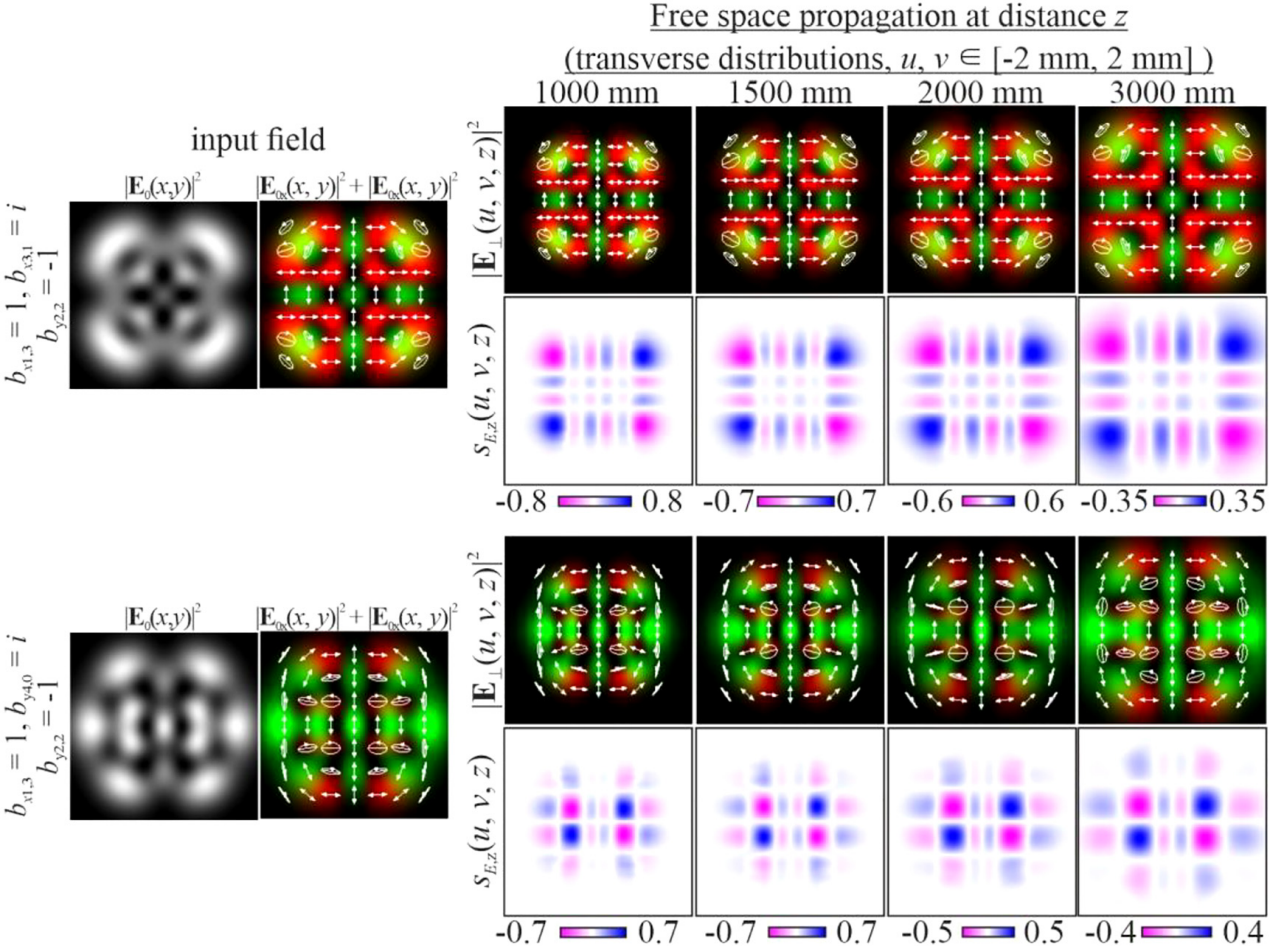
Propagation of nonuniformly polarized multimode HG beams defined by Eq. (11) with indices satisfying the condition defined by Eq. (12) (nonuniformly polarized vector modes).

**Figure 8:**
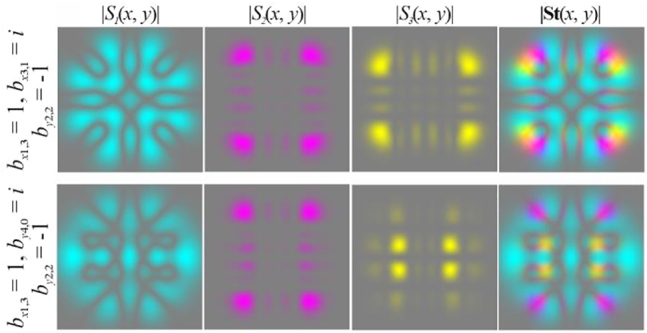
Distribution of the Stokes vector defined by Eq. (24) for inhomogeneously polarized beams presented in Figure 7.

Obviously, by satisfying the condition defined by Eq. (12) for several (more than two) modes, it is possible to form more complex vector modes described by Eq. (11). In particular, Figure 6 shows the simulation results for a circularly polarized beam, and Figure 7 shows examples for nonuniformly polarized fields consisting of HG modes with indices from Ω:n+m=4. For nonuniformly polarized fields, Figure 8 shows the corresponding distributions of the amplitudes of the Stokes vector components defined by Eq. (24) in the input plane. It can be seen that the state of polarization may be very complex.

As follows from the simulation results, the proposed method enables the formation of vector modes with a wide variety of intensity distributions and polarization states.

## 4 Experimental results

**Figure 9:**
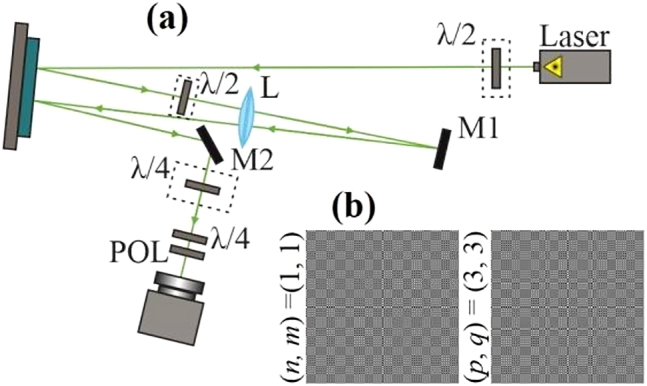
(a) Optical setup for the generation and investigation of the designed uniformly polarized and inhomogeneously polarized vector superpositions of HG modes. Laser is a solid-state laser (*λ* = 532 nm), SLM is the spatial light modulator HOLOEYE PLUTO VIS (1920 × 1080 pixels, with a pixel pitch of 8 μm), L is a lens with a focal length of 350 mm, M1 and M2 are mirrors, POL is a polarizer-analyser, *λ*/2 and *λ*/4 are half- and quarter-wave plates, and CAM is a video camera. (b) Examples of the encoded phase masks used in the experiments.

**Figure 10:**
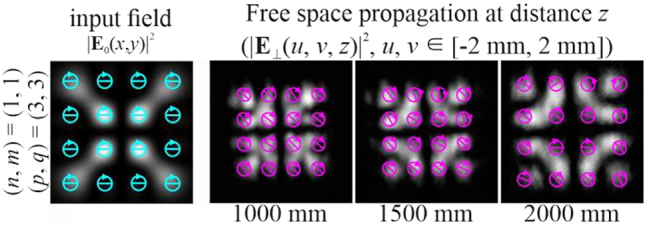
Experimental results for a “+”-circular polarization two-mode HG beam defined by Eq. (26) with indices (*n* + *m*) ≠ (*p* + *q*).

To generate the designed uniformly polarized and inhomogeneously polarized vector superpositions of HG modes, we used the well-known interferometric method (see Figure 9). Using a first half-wave plate, the polarization direction of the linear polarization of a horizontally polarized Gaussian laser beam from a Nd:YAG laser with a wavelength of 532 nm was rotated by 45°. Then, the laser beam was directed toward a display of the reflective spatial light modulator (SLM) HOLOEYE PLUTO VIS (1920 × 1080 pixels, with a pixel pitch of 8 μm). The SLM is optimized only for the modulation of a horizontally polarized beam, so after the first reflection, only the horizontally polarized component of the laser beam is modulated, while the vertically polarized component of the laser beam remains nonmodulated. The second half-wave plate rotates the polarization of the reflected laser beam by an angle of 90°. A mirror M1, located in the back focal plane of a lens L with a focal length of 350 mm, was used for the reflection of the generated laser beam and for redirecting it to the SLM display located in the front focal plane of the lens L. After the second reflection from the SLM, the component of the laser beam that was nonmodulated after the first reflection is now modulated. Thus, after the second reflection, the addition of two laser modes generated by different phase masks is observed. The laser beam that was reflected a second time from the SLM was directed to a video camera CAM mounted on an optical rail using a mirror M2. In the case of the generation of uniformly polarized laser beams (i.e. circularly polarized superpositions of HG modes), the half-wave plates were removed from the optical scheme, and a quarter-wave plate QP1 was introduced in the path of the generated laser beam after a second mirror M2. For the generation of inhomogeneously polarized three-mode HG vector beams, the first phase mask displayed in the SLM was a phase mask of the *x*-polarized mode or a combination of two modes in superposition, and the second displayed phase mask was a phase mask of the *y*-polarized mode or a combination of two modes in superposition. The amplitude encoding was used for the implementation of pure-phase masks from initial amplitude-phase functions [76]. Then, for the recording of the Stokes parameters, we used a combination of a quarter-wave plate and a polarizer POL [77]. For some cases of the designed laser beams, the experimentally measured intensity distributions with the recovered polarization maps are shown in Figures 10 [Fig j_nanoph-2021-0418_fig_011] [Fig j_nanoph-2021-0418_fig_012] [Fig j_nanoph-2021-0418_fig_013] [Fig j_nanoph-2021-0418_fig_014]–15. There is a good agreement between the experimental and the modeled results.

**Figure 11: j_nanoph-2021-0418_fig_011:**
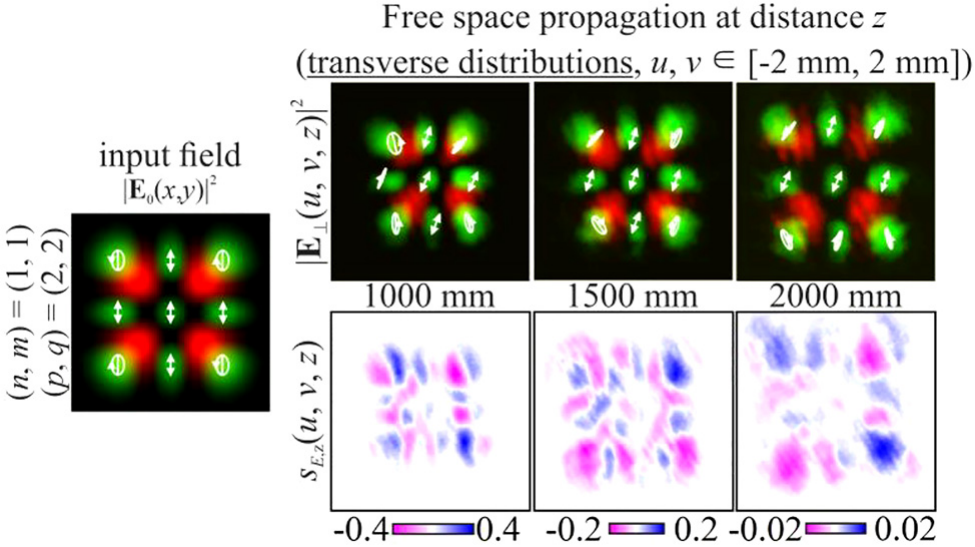
Experimental results for an inhomogeneously polarized two-mode HG beam defined by Eq. (27) with indices (*n* + *m*) ≠ (*p* + *q*).

**Figure 12: j_nanoph-2021-0418_fig_012:**
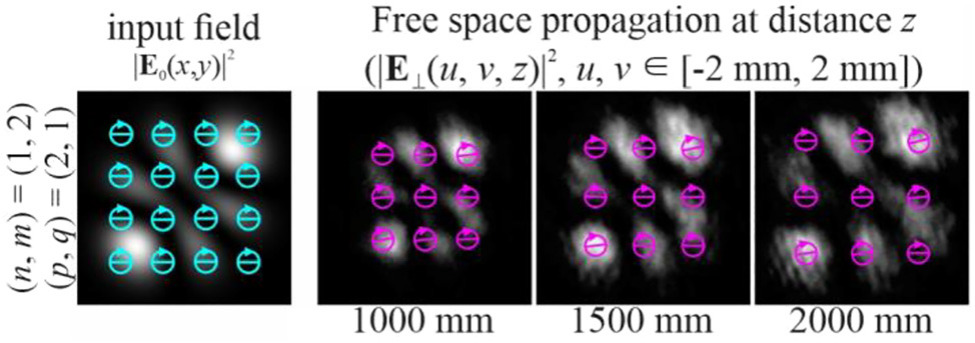
Experimental results for a “+”-circular polarization two-mode HG beam defined by Eq. (26) with indices (*n* + *m*) = (*p* + *q*) (a uniformly polarized vector mode).

**Figure 13: j_nanoph-2021-0418_fig_013:**
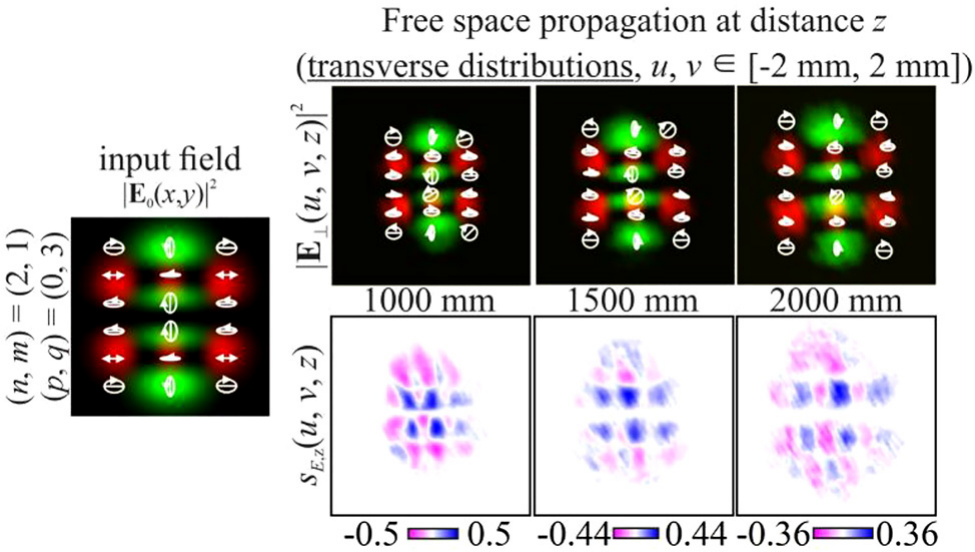
Experimental results for an inhomogeneously polarized two-mode HG beam defined by Eq. (27) with indices (*n* + *m*) = (*p* + *q*) (a nonuniformly polarized vector mode).

**Figure 14: j_nanoph-2021-0418_fig_014:**
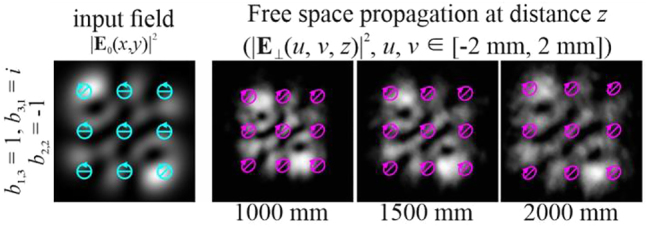
Experimental results for a circularly polarized multimode HG beam defined by Eq. (11) with indices satisfying the condition defined by Eq. (12) (a uniformly polarized vector mode).

**Figure 15:**
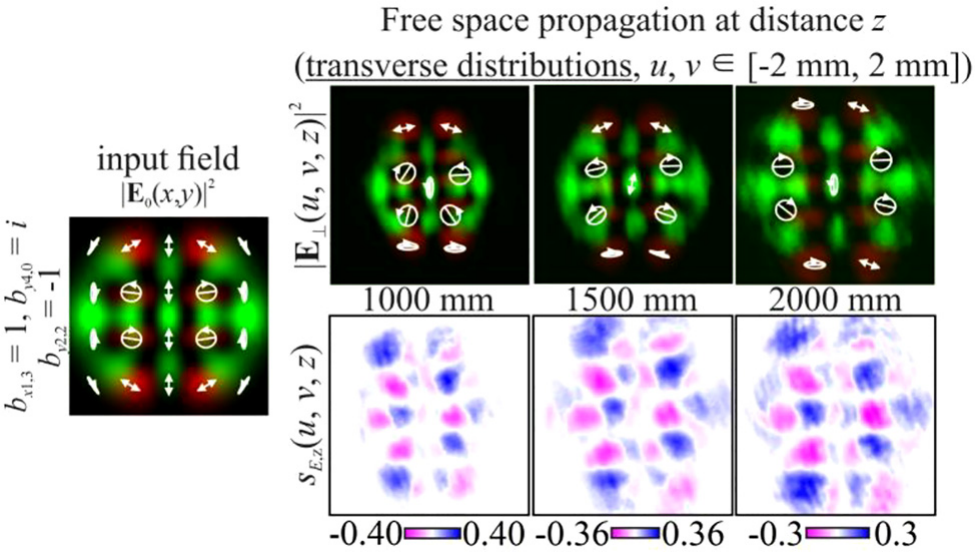
Experimental results for an inhomogeneously polarized multimode HG beam defined by Eq. (11) with indices satisfying the condition defined by Eq. (12) (a nonuniformly polarized vector mode).

For uniformly polarized beams with circular polarization, the SAM density distribution $s_{E,z}\left(u,v,z\right)$ coincides with the intensity distribution of the field ${\left|E_{⊥}\left(u,v,z\right)\right|}^{2}$ (Figures 10, 12, and 14). For nonuniformly polarized beams, we used the direct correspondence between the distribution of $s_{E,z}\left(u,v,z\right)$ and the Stokes parameter $S_{3}\left(u,v,z\right)$ (23), which was experimentally measured (Figures 11, 13, and 15). The digits on the scale bars are given in accordance with the normalization to the global maximum value in the considered 3D region: u,v∈[−2 mm, 2 mm] , z∈[0, 2000 mm] .

As can be seen from Figure 10, when (n+m)≠(p+q) , the stability or conservation of the distribution $s_{E,z}\left(u,v,z\right)$ is not ensured during the propagation of the beam, although the polarization state remains approximately the same throughout the space. From the results shown in Figure 11, it follows that for (n+m)≠(p+q) , the polarization state changes significantly. In particular, at a distance *z* = 2000 mm, practically the entire field becomes linearly polarized, although regions with circular and elliptical polarization occur in the input field (*z* = 0). Therefore, both the absolute values of $s_{E,z}\left(u,v,z\right)$ and the total SAM value decrease with increasing distance.

As can be seen from Figure 12, when (n+m)=(p+q) , the invariance (up to scale) of the intensity distribution and polarization state, as well as $s_{E,z}\left(u,v,z\right)$ , is ensured during the propagation of the beam. Thus, the formation of a uniformly polarized vector mode is shown.

From the results shown in Figure 13, it can be seen that for (n+m)=(p+q) , an approximate preservation (up to scale) of a rather complex inhomogeneous initial state of polarization is provided. Some of the errors are associated with experimental measurements. Note that the absolute values decrease only because of scale broadening, i.e. in full accordance with the simulation results.

Figures 14 and 15 show the experimental results of the formation of more complex vector modes with both uniform (Figure 14) and nonuniform (Figure 15) polarization.

The proposed visualization of the Stokes vector makes it possible to clearly and in detail to see the polarization features of the beams under study. This is especially convenient for experimental results. In particular, for experimentally generated nonuniformly polarized vector modes (see Figures 11, 13, and 15), the patterns of the intensity distribution and the polarization state are supplemented by the patterns of the SAM longitudinal component distribution which is proportional to the third Stokes parameter. So, it is possible to form rather complex SAM distributions containing regions with different spin values and directions. Microparticles trapped in these local areas will experience the corresponding effect of SAM, i.e. rotate at different speeds and directions. Also, this SAM distribution can be used for complex laser surface structuring. Moreover, these distributions rather well (some errors are associated with experimental implementation) retain their structure during beam propagation, which facilitates beam positioning during laser processing, as well as during optical trapping and micromanipulation.

Thus, we have shown that it is possible to generate beams whose main field characteristics (intensity, polarization state, SAM density) remain structurally invariant during beam propagation, even for very complex distributions.

## 5 Conclusions

We investigated the generation of vector modes based on inhomogeneously polarized HG vector beams, providing complete structural conservation of the beams during propagation. Like uniformly polarized mode beams, these beams provide structural stability (or invariance) of both the intensity and the polarization state, in turn ensuring the stability (or conservation) of other field characteristics, including the angular momentum.

To generate HG vector modes, the transverse components of the input electromagnetic field should contain HG modes with indices whose sum is the same. For the visual analysis of the polarization state of inhomogeneously polarized beams, we use the transverse distribution of the vector of three Stokes parameters. Moreover, the correspondence of the third Stokes parameter to the distribution of the longitudinal component of the SAM is used for experimental measurements.

Simulation and experimental results have shown that it is possible to generate beams whose intensity, polarization, and SAM are structurally stable, even for complex distributions. This expands the possibilities of using structured laser beams in various applications. The SAM is contributed to both optical force and torque exerted on nano and microspheres. So, the appearance of the longitudinal and transverse SAM in the designed beams can be transferred to nano and microparticles which will produce a torque on the optically trapped particles and provide additional rotation degrees of freedom for optical manipulation applications [78]. In addition, such complex laser beams can be used for optically controlled spin transfer for the creation and mapping of a spin current in materials [32]. The control of spin current in semiconductors has been considered for over 20 years as crucial for the development of new data storage and implementation of a quantum computer [79, 80]. Recently, it was shown that structured light can be applied to interfering photoexcitation pathways in gallium arsenide to sculpt the spatial and momentum configuration of its conduction band population [81]. It should be noted that the generation structurally stable laser beams with the desired SAM density potentially can be used for the implementation of SAM-carrying optical communications transferred information signals encoded in the distribution of SAM density like in the case of optical communications based on OAM laser beams with SAM [82] or higher-order cylindrical vector beams (CVBs) [83].
